# Quantifying potential fluid transfused through pressure monitoring and circuit flushes in pediatric ECMO patients

**DOI:** 10.1051/ject/2024007

**Published:** 2024-06-18

**Authors:** Steven Robertson, Katherine White

**Affiliations:** Children’s Heart Center of Nevada Las Vegas NV USA

**Keywords:** Neonate, Pediatric, ECMO, Pressure Transducer, Pressure Monitoring

## Abstract

Pressure monitoring on pediatric Extracorporeal Membrane Oxygenation (ECMO) circuits is used to aid in the evaluation of patient hemodynamics and circuit health. Extracorporeal Life Support Organization (ELSO) recommends monitoring pressures on the venous line, pre-, and post-oxygenator. In order to keep pressure ports patent, crystalloid can be used as a flush. The fluid transfused to the patient through these lines can be challenging to quantify accurately due to variance in clinician practice. Currently, there is no published data or practice suggestions on this topic. In Vitro experiments using Edwards True Wave transducers and pressure bags were constructed, allowing for common negative and positive pressures to be simulated. Passive volume infused through the transducer as well as intermittent active flushing by pulling the snap tab were measured and the volumes were recorded. When the pressure transducer and associated tubing are kept patent by using a pressurized IV bag, per the instructions for use, the daily volume transfused was found to be 319.6 mL or close to a typical neonate’s total blood volume. Rather than using passive or active flushing, the use of automated syringe pumps can reduce the transfused volume to 24 mL per day. Further study is recommended to develop and publish best practices.

## Overview

Extracorporeal Membrane Oxygenation (ECMO) is a well-established, life-saving therapy for critically ill patients in the neonate and pediatric populations who are in cardiac or respiratory distress [1]. Even though it is not an industry standard, some form of pressure monitoring has become a common practice with ECMO in adherence with the Extracorporeal Life Support Organization (ELSO) literature [2]. According to a 2011 survey involving ELSO centers, negative pressure monitoring on the venous line was used by 83% of neonatal ECMO programs using centrifugal pumps [3]. Negative pressure from the venous cannula and positive pre-membrane and post-membrane oxygenator pressures can be monitored in order to aid in the evaluation of circuit health [4]. This includes but is not limited to, measuring circuit pressure in order to determine the pressure drop across the oxygenator, clot formation, and the siphon effect in the venous line. ECMO circuit pressures are also frequently monitored in order to evaluate patient hemodynamics, volume status, and they function as safety devices.

Circuit pressure monitoring is handled in a myriad of ways across the world. ECMO disposables like Cardiohelp (Getinge, Rastatt, Germany) use internal pressure monitoring that does not require flushing. While this device is intended for the adult population, off-label use in pediatrics has been studied and deemed safe [5]. Some centers refrain from pressure monitoring entirely in an effort to simplify circuits, thus reducing the chances of line separations, air entrainment, and blood spillage. Although not well documented outside of institutional protocols, it is an accepted practice to flush external pressure transducers frequently with fluid in order to reduce areas of stagnation and keep pressure monitoring ports patent. This practice aids in preventing the development of thrombus formation which can lead to an adverse event in the mechanical components, or even a sentinel patient event [6]. However, this protective measure can also contribute to fluid overload in the pediatric patient [7]. Because of this and the safety concerns listed previously, some centers choose to simply not flush transducers in any capacity.

ECMO pressure transducers may be flushed in a number of ways. Syringe and IV pumps can be used to supply constant rates of flush-through transducers to circuit connection points. A crystalloid bag pressured to 300 mmHg can be used to passively flush through the transducer as well as manually by pulling on the snap tab. Similarly, a crystalloid-filled syringe may be manually pushed with a desired amount of volume at the pressure ports.

Circuit flushes may be passive, active, or both. Passive flushing is allowing the volume to gradually pass into the pressure lines and ports without intervention. This is done using a pressurized bag set at a desired pressure. Active fluid flushing is the act of manually pulling the transducer flush tab or pushing volume directly into the circuit with a fluid-filled syringe for a period of time. The amount of fluid transfused passively and actively through pressure transducers is highly variable due to pressure differences in the pediatric ECMO circuit. Likewise, the fluid used during active pressure transducer flushes via the snap tab may be inconsistent amongst clinicians based on personal preference, hemodynamics, or current circuit conditions.

The purpose of this investigation is to evaluate the quantity and variability in volume measured across the flush device in pressure transducers depending on resistance. This would provide clinicians with a more accurate daily fluid balance and ultimately reduce patient fluid overload while on ECMO.

## Description

In order to quantify the amount of volume transfused through pressure transducers in pediatric ECMO circuits, six experiments were performed. The first three were designed to measure passive flushing through the Edwards TruWave transducer with blue Snap-Tab (Edwards Lifesciences Corporation, Irvine, CA, USA) against three different pressure conditions: 0 mmHg, 150 mmHg, and −50 mmHg. The final three experiments were to quantify the amount of volume actively transfused while pulling on the Snap-Tab of the pressure transducer flushing device.

### Experiment 1 (0 mmHg)

**Figure 1.**
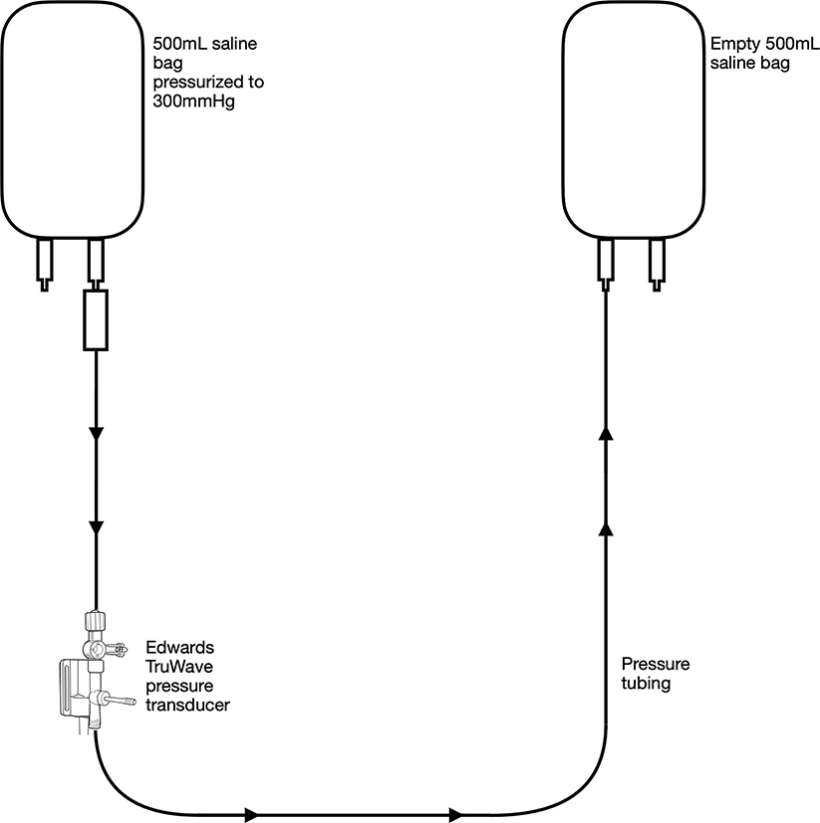
In Vitro setup for Experiments 1 and 4 simulating volume transfused from a pressurized bag of saline, through a pressure transducer, to an empty saline bag.

As illustrated in Figure 1 a 500 mL bag of 0.9% saline (Baxter Healthcare Corporation, Deerfield, IL, USA) was pressurized to 300 mmHg using an Ethox 500 mL pressure infusion bag (Ethox Medical, LLC, Grand Rapids, MI, USA). The pressure bag was continually monitored throughout the experiment and kept at 300 mmHg utilizing the green 300 mmHg indicator. The saline bag was spiked using an Edwards TruWave 3 cc/VAMP 7″ pressure monitoring set (Edwards Lifesciences Corporation, Irvine, CA, USA). The tubing below the transducer was discarded and a pressure line was added. The opposite end was connected to a BD MaxGuard bag access spike (Becton, Dickinson and Company, Lakes, NJ, USA). An empty 500 mL 0.9% saline bag was spiked and all lines were primed using the Snap-Tab flushing device. This empty bag was hung at the same level as the pressurized bag. After 24 h, the passively shunted volume from the pressurized saline bag to the empty bag was removed and measured using a 50 mL syringe.

### Experiment 2 (150 mmHg)

**Figure 2.**
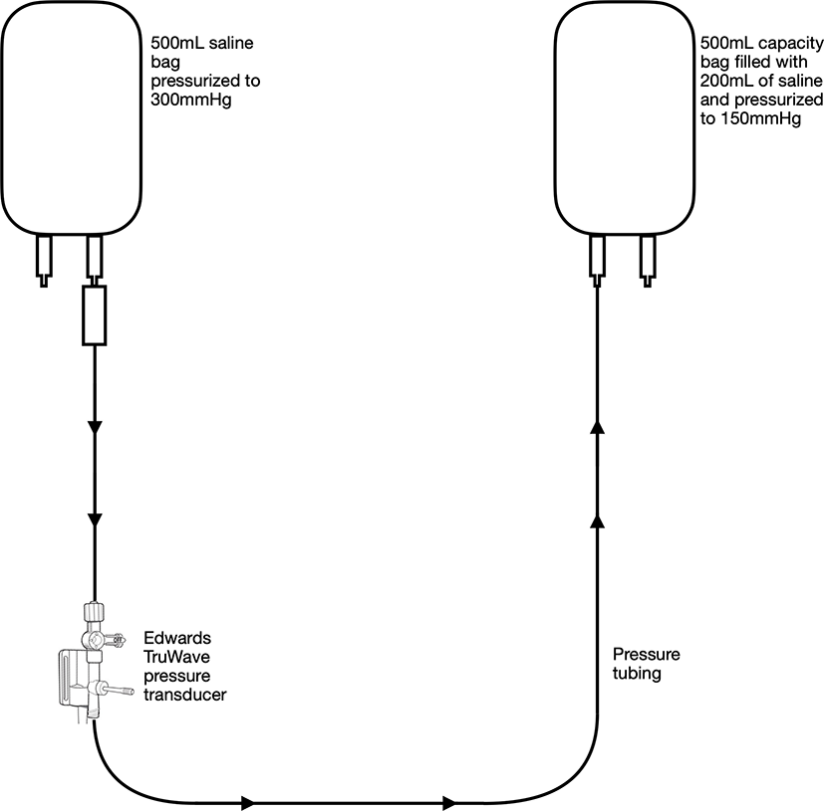
In Vitro setup for Experiments 2 and 5 simulating volume transfused from a pressurized bag of saline, through a pressure transducer, to a bag full of 200 mL of saline and under 150 mmHg of pressure.

Experiment 2 was set up the same as experiment 1 with the following modifications: the empty 500 mL saline bag was filled with 200 mL of 0.9% saline, placed into an Ethox 500 mL pressure infusion bag, and pressurized to 150 mmHg (Figure 2). The pressure bag was continually monitored throughout the experiment and kept at 150 mmHg utilizing the white 150 mmHg indicator. After 24 h the 150 mmHg bag was depressurized and the initial 200 mL 0.9% saline was removed. The remaining 0.9% saline was removed and measured.

### Experiment 3 (−50 mmHg)

**Figure 3.**
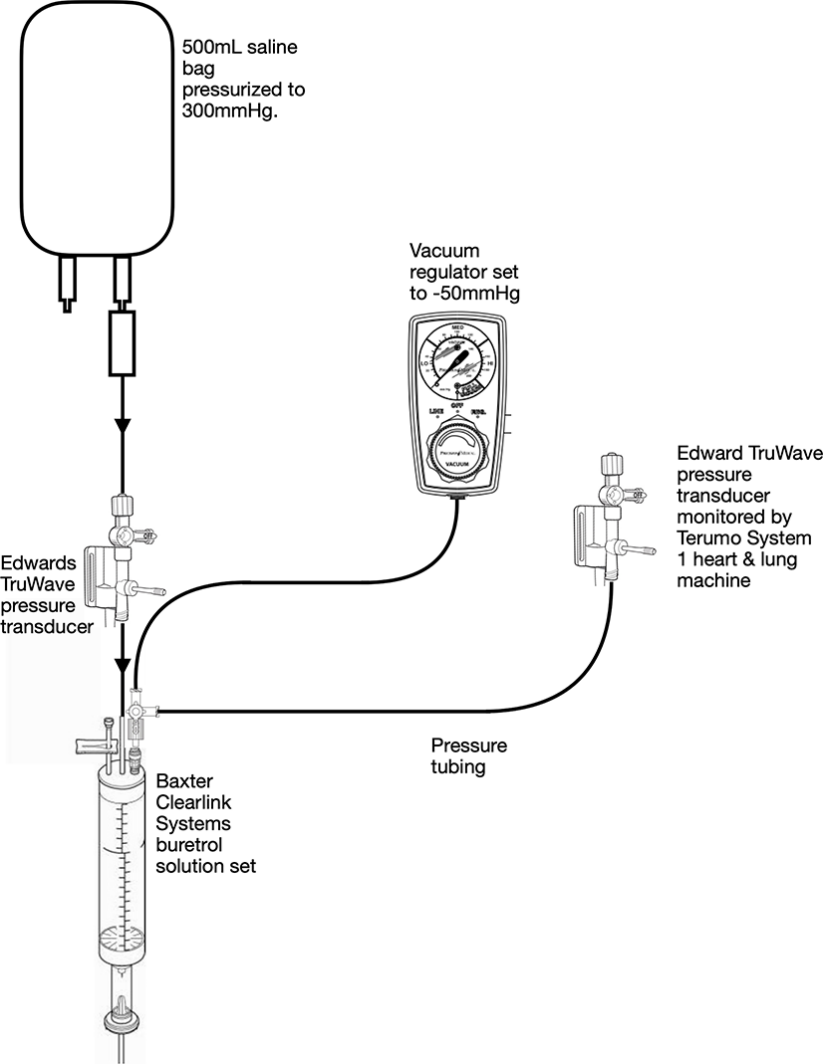
In Vitro setup for Experiments 3 and 6 simulating volume transfused from a pressurized bag of saline, through a pressure transducer, to an empty buretrol solution set with negative 50 mmHg of vacuum applied.

Experiment 3 was set up the same as experiment 1 with the following modifications: the empty saline bag was removed and replaced with a Baxter Clearlink Systems buretrol solution set (Baxter Healthcare Corporation, Deerfield, IL, USA), a three-way stopcock was attached to the luer connector on the top of the buretrol solution set, and a pressure line was connected to a vacuum regulator with the opposite end attached to the stopcock (Figure 3). Another pressure line was added to the remaining port of the stopcock with the opposite end connected to an Edwards TruWave transducer monitored by a Terumo System One heart and lung machine (Terumo Cardiovascular, Ann Arbor, MI, USA). The vacuum regulator was turned on and the pressure inside the buretrol solution set was regulated to −50 mmHg. After 24 h the volume was removed and recorded.

Experiments 4, 5, and 6 were set up the same as experiments 1, 2, and 3, respectively. In these trials, the Snap-Tab was pulled for 10 s and the expelled fluid volume was recorded. Ten seconds was selected as a time that was reliably repeatable and provided a clearly measurable amount of volume to report. All experiments were repeated five times.

The TruWave pressure transducer with blue Snap-Tab displaces 3 mL ± 1 mL passively every hour across the internal flush device when connected to an IV bag pressurized to 300 mmHg [8]. Experiment 1 results confirm this claim (Table 1). The findings from experiments 2 and 3 allow clinicians to extrapolate how much volume is passively transfused to patients per hour or per day through pressure transducers connected to the ECMO circuit based on different circuit pressures (Table 1). The final three experiments quantify how much volume can be infused while actively flushing pressure transducers against common circuit pressures by taking note of how many seconds the clinician pulls on the Snap-Tab (Table 2).

**Table 1:** Pressure transducer passive infusion volume (mL) over 24 h period.

Experiment 1 (300 mmHg to 0 mmHg)				
Test 1	Test 2	Test 3	Test 4	Test 5
68	75	75	72	71
		Average: 72.2 mL/day or 3.00 mL/h		
Experiment 2 (300 mmHg to 150 mmHg)				
Test 1	Test 2	Test 3	Test 4	Test 5
41	43	38	40	44
		Average: 41.2 mL/day or 1.72 mL/h		
Experiment 3 (300 mmHg to −50 mmHg)				
Test 1	Test 2	Test 3	Test 4	Test 5
91	89	91	87	87
		Average: 89.0 mL/day or 3.71 mL/h

**Table 2:** Pressure transducer active infusion volume (mL) over 10 s.

Experiment 4 (300 mmHg to 0 mmHg)				
Test 1	Test 2	Test 3	Test 4	Test 5
29	30	28	30	29
		Average: 2.92 mL/s		
Experiment 5 (300 mmHg to 150 mmHg)				
Test 1	Test 2	Test 3	Test 4	Test 5
41	43	38	40	44
		Average: 1.98 mL/s		
Experiment 6 (300 mmHg to −50 mmHg)				
Test 1	Test 2	Test 3	Test 4	Test 5
91	89	91	87	87
		Average: 3.86 mL/s		

## Discussion

The experiments outlined above provide evidence that the fluid transfused to ECMO patients through circuit pressure transducers can be significant when using an Edwards TruWave transducer and pressurized IV bag. Passive volume transfused daily to a patient using the data from Table 1 would equal 154.6 mL, assuming three transducers reading pressures on the venous line as well as pre and post-oxygenator respectively. This is using the conservative values from the 0 mmHg experiment for the venous line and 150 mmHg for both of the oxygenator transducers. Anticipating a 1-second pull per hour on all transducer snap tabs, an additional 165.1 mL would be actively transfused in the same 24-hour period. Combining both passive and active values shows that 319.6 mL would be transfused. While these findings may not be clinically significant in the adult population, in a neonate, this can equate to nearly their entire circulating blood volume in crystalloid flush alone [9]. Excessive crystalloid infusion of this nature is known to exacerbate acute inflammatory response and lead to the third spacing of fluid [10].

The tests performed also reveal the total volume given is difficult to calculate with 100% accuracy. Different variables such as constantly changing circuit pressures, as well as clinician practice, yield a variety of outcomes. For example, a patient requiring higher flows might have a higher negative pressure on the venous line, resulting in more passive volume transfused. Compare that to an aging oxygenator having a higher positive pressure on the pre oxygenator line, resulting in less volume given. Also, a clinician on the morning shift might only pull the snap tab for half a second each hour, leading to less volume transfused compared to their night shift counterpart who pulls for over 2 s out of an abundance of caution, resulting in more volume transfused. For these reasons, IV bags pressurized to 300 mmHg should not be considered best practice. As an alternative, syringe pumps can be used to deliver a constant infusion through the pressure transducers. With this proposed method, active flushing is eliminated and the crystalloid volume can be accurately captured lending to a more precise daily fluid balance. The infusion can also be titrated to the location of each pressure port.

As a result of the findings from these experiments, pressurized IV bags were removed from practice and syringe pumps were adopted as part of the institutional protocol to use for flushing pediatric ECMO circuit pressure transducers. The pumps were set at 0.5 mL/h on the pre-membrane, 0.3 mL/h on the post membrane, and 0.2 mL/h on the venous return port. This equates to a total volume of 1 mL per hour or 24 mL within a 24-hour period. The most noted advantage of the syringe pump is that the total volume transfused can be drastically reduced, thus reducing fluid overload on ECMO, and yielding improved patient outcomes [11]. Additionally, syringe pumps hold an advantage over IV pumps as the syringes provide a visual of volume transfused when device precision is in question [12].

Due to the immense variation in practice, it is proposed that a survey be completed to discover all the methods being used to monitor ECMO pressures as well as maintaining patency. Additionally further studies need to be done and guidelines should be developed by ELSO in order to standardize the practice of monitoring and flushing pressure ports on ECMO.

## Data Availability

All available data are incorporated into the article.
